# Federated learning’s uncomfortable truth: why human networks matter more than neural networks

**DOI:** 10.1093/jamia/ocag047

**Published:** 2026-04-15

**Authors:** Laura-Maria Peltonen, Taridzo Chomutare

**Affiliations:** Department of Health and Social Management, University of Eastern Finland, Yliopistonranta 8, P.O. Box 1627, FI-70211 Kuopio, Finland; Faculty of Medicine, University of Turku, Kiinamyllynkatu 10, Turku 20520, Finland; Turku University Hospital, Kiinamyllynkatu 4–8, Turku 20520, Finland; Kuopio University Hospital, Puijonlaaksontie 2, Kuopio 70210, Finland; Technology and Artificial Intelligence Department, Norwegian Centre for E-health Research, University Hospital of North Norway, Postbox 100, Tromsø 9038, Norway

**Keywords:** federated learning, data governance, privacy, health information exchange, interinstitutional relations

## Abstract

**Objectives:**

To examine real-world barriers to implementing federated learning in healthcare and highlight the organizational, regulatory, and socio-technical factors often overlooked in technical research.

**Materials and Methods:**

Insights were derived from a 3-year implementation of a Nordic–Baltic federated health data network involving 5 countries and 9 institutions, incorporating legal, organizational, and cross-disciplinary perspectives.

**Results:**

Structural challenges included coordination burdens, divergent interpretations of privacy and risk, epistemological gaps between disciplines, and the absence of legal frameworks for multi-country distributed learning in Europe. These constraints limited progress despite the availability of robust technical solutions.

**Discussion:**

Technical privacy measures alone cannot replace trust-building, governance development, and cross-disciplinary translation work. Federated learning is more accurately understood as a socio-technical collaboration model rather than a purely technical architecture.

**Conclusion:**

Pre-implementation planning, tiered participation models, and strengthened governance are essential to support equitable, sustainable, and clinically impactful adoption of federated learning in healthcare.

## Introduction

The *Nordic-Baltic Federated Health Data Network* spent 3 years discovering what the field doesn’t want to admit: federated learning’s challenges aren’t primarily technical; they are human. After implementing a cross-border network across 5 countries and 9 institutions, we found that success depends far more on governance frameworks, trust-building, and interdisciplinary translation than on algorithmic sophistication. This commentary challenges the prevailing techno-optimism surrounding federated learning and offers a path forward based on implementation reality rather than theoretical promise.

The FederatedHealth project (2022-2025), funded by Nordic Innovation, brought together 9 institutions across 5 Nordic–Baltic countries (Finland, Sweden, Norway, Denmark, and Estonia) to develop a federated health data network for clinical natural language processing. The consortium included university hospitals, research centers, and e-health organizations, with teams spanning clinical medicine, nursing science, computer science, linguistics, law, and ethics. Built on NVIDIA FLARE, the network aimed to train multilingual clinical large language models on distributed electronic health record data, targeting detection of medical implants and identification of adverse drug reactions from unstructured clinical text.^1^^,^^2^ To be clear, this commentary is not an argument against federated learning or in favor of centralized data models. Centralized approaches carry well-recognized limitations, including heightened privacy risks, single points of failure, and barriers to cross-institutional participation. Rather, we argue that realizing federated learning’s potential requires honest engagement with its non-technical challenges, challenges that are currently underrepresented in the literature.

## The implementation delusion

The federated learning literature suffers from a fundamental disconnect. Of 612 recent healthcare studies, only 5.2% involve real-world clinical applications.^3^ The rest exist in a parallel universe of perfect data, willing partners, and frictionless collaboration. This isn’t advancing the field; it’s creating an echo chamber of technical solutions to human problems.

Our experience implementing FederatedHealth revealed 3 uncomfortable truths for the field to confront:

1. *Federated learning multiplies rather than reduces complexity*
  The promise of “*keeping data local*” obscures a reality where every participating site needs expertise to execute, debug, and validate federated jobs. What takes one engineer in a centralized system requires multiple coordinated teams in our federated approach. Firewall configurations alone consume weeks. The coordination overhead is not a bug to be fixed but an inherent feature of distributed systems involving multiple sovereign entities.
2. *Privacy preservation is necessary but insufficient*
  While federated learning elegantly solves the data movement problem, it may create new vulnerabilities, model inversion attacks, gradient leakage, and membership inference, which many implementations may inadequately address. More critically, the assumption that technical privacy guarantees translate to institutional trust is naive. We spent months developing a *common model* and *code of conduct*, not because the technology required it, but because human organizations needed formal frameworks to manage risk and liability.
3. *The knowledge translation challenge dwarfs the technical challenge*
  When a data scientist says “*differential privacy*,” a clinician thinks “*patient confidentiality*,” and a lawyer hears “*GDPR—Article 89*.” These aren’t synonyms, but different concepts with different implications. Our pre-implementation workshops revealed that even basic terms like “*data quality*” had no shared meaning across disciplines.^2^^,^^3^ This is not a communication problem alone; it is an epistemological divide that requires active, ongoing translation work.

## The governance gap nobody wants to discuss

Papers celebrating federated learning’s “*privacy-preserving*” architecture rarely acknowledge a fundamental gap: Europe lacks a legal framework for multi-country health data networks.^4^ The GDPR was not designed for distributed learning, and national interpretations differ substantially.^5^ Current workarounds, such as joint controllership with distributed responsibilities are less true innovation than pragmatic compromises, fragile constructs that hold only until tested. Nonetheless, federated architectures may still offer a viable pathway toward a pan-European data ecosystem. Yet the upcoming European Health Data Space (EHDS),^6^ while promising regulatory clarity, risks reinforcing the very “*centralized data silos*” it aims to dismantle, thereby raising privacy and security concerns.^7^ Moreover, a supportive EU environment does little to resolve challenges in global collaborations. A further unresolved issue is ownership of collaboratively trained models: who holds rights to a model trained on data from 6 institutions across 5 countries? Current proposals either ignore intellectual property questions altogether or suggest simplistic solutions unlikely to withstand scrutiny by technology transfer offices.

## The resource reality check

Looking at costs, something the literature systematically underreports:

1. *Human infrastructure*: The majority of our budget needs to be allocated to coordination, governance and knowledge translation activities, which are typically dismissed as “*overhead*,” but are actually foundational to these kinds of projects.
2. *Technical disparities*: Partner sites may range from single GPUs to larger clusters, creating a 2-tier system where resource-rich institutions may end up dominating.
3. *Hidden multiplicators*: Every technical decision requires multi-way negotiation with several partners, every debugging session needs local expertise and every model update needs a security review.

The federated learning literature’s failure to acknowledge these cost implications is misleading, and it sets up projects for failure. When institutions budget for federated learning based on published studies, they are planning for a marathon with sprinter’s provisions. Recent reviews confirm that most federated learning implementations are “*not appropriate for clinical use*” due to methodological flaws, including privacy concerns, generalization issues, and communication costs, that burden them.^8^

## Reconceptualizing federated learning: from technology to socio-technical systems

Having outlined the core challenges encountered in our implementation, we now turn to strategies that emerged from our experience and that we believe can guide future federated learning initiatives. The path forward requires a fundamental reconceptualization. Federated learning is not a technical solution that happens to have organizational challenges. It is an organizational collaboration method that happens to use technical infrastructure. This is a paradigm shift with practical implications. Encouragingly, several established networks already demonstrate that this reconceptualization works in practice. The Observational Health Data Sciences and Informatics (OHDSI) community^9^ and the European Health Data & Evidence Network (EHDEN)^10^ have achieved large-scale federated collaboration, with EHDEN harmonizing over 210 data sources across 30 countries,^11^ by investing heavily in community infrastructure, including education programs, shared training materials, regular workshops and conferences, and the development and maintenance of common data models such as OMOP. Their success was built not on algorithmic innovation alone but on sustained investment in the human networks that enable technical collaboration.

### Pre-implementation: where real success is determined

Our most valuable contribution is not our technical implementation but a pre-implementation methodology. Before writing a single line of code, we suggest:

1. Map the actual competency landscape in your team, using structured self-assessments, revealing disparities in expertise, which are greater barriers than disparities in data.
2. Use participatory design methods to surface hidden assumptions, conflicting priorities and agree on shared goals and solutions.
3. Develop translation frameworks for interdisciplinary communication, creating shared vocabularies before shared models.
4. Establish governance before algorithms, recognizing that trust networks must precede neural networks.
5. Be mindful of sustainability, understood as ensuring that solutions are:

 • Clinically relevant (fit for purpose and appropriate to context)
 • Ethically defensible (addressing privacy, fairness, and data balance)
 • Socially legitimate (acceptable and implementable in real-world practice)
 • Economically viable (affordable and feasible to maintain)
 • Environmentally responsible (minimizing ecological impact)

This pre-work, typically absent from federated learning projects, predicted our implementation challenges with remarkable precision. The barriers identified in workshops, security concerns, infrastructure heterogeneity, and coordination complexity manifested as anticipated.^2^

## Tiered participation: embracing rather than hiding inequality

The federated learning ideal assumes that equal partners contribute equally. However, reality features radical disparities in resources, expertise, and commitment. Rather than pretending these do not exist, we propose a tiered participation model:

*Tier 1*: Full partners hold comprehensive multidisciplinary expertise (technical, domain, legal, ethical, and social); technical capacity; and data resources, enabling them to contribute across all aspects of the project.

*Tier 2*: Contributing partners possess only some of these elements and therefore focus on selected tasks, perspectives, or goals, supported by full partners where needed.

*Tier 3*: Specialist partners provide specific expertise, infrastructure, or datasets. Their contribution is more focused and requires integration and support from full partners.

This model does not compromise the federated ideal, but it makes it achievable. Requiring identical capabilities from all partners would ensure that only resource-rich institutions can participate, creating the very centralization that federated learning is meant to prevent. Instead, the tiers are mutually reinforcing; where one group lacks a capability, another can fill this gap. However, interconnections between the different actors need careful negotiation to work effectively, and roles, responsibilities, expectations, and support needs addressing to ensure fairness, transparency, and impact.

## Regional before global: the uncomfortable geopolitics

The federated learning literature envisions global collaborations, conveniently ignoring that data sovereignty is not just technical but deeply political. Our *Nordic-Baltic focus* was not arbitrary. It leveraged existing trust relationships, similar regulatory frameworks, and cultural alignment that made collaboration possible. The field needs to acknowledge that successful federated networks will likely be regional, aligned with geopolitical realities rather than technical capabilities. A European federation, an Asian federation, an American federation—these make sense. A global federation remains aspirational, blocked less by technology than by trust.

We acknowledge that our experience is situated within the European regulatory context, where the GDPR and divergent national implementations create specific coordination challenges. In other contexts, such as the United States, where HIPAA provides the uniform federal framework, or in low- and middle-income country settings, where regulatory infrastructure may be less developed, the specific regulatory barriers will differ. However, we contend that the core socio-technical challenges we identify, the need for trust-building, governance development, interdisciplinary translation, and realistic resource planning, transcend any single regulatory environment and apply wherever multiple institutions attempt federated collaboration.

## The interdisciplinary imperative

Perhaps our most striking finding was how differently stakeholders conceptualized the same system. Clinicians saw federated learning as a means to access broader patient populations. Computer scientists saw it as a distributed optimization problem. Lawyers saw it as a liability distribution mechanism. Ethicists saw it as a test of fairness, questioning whether federated learning protects privacy, ensures equity, and sustains trust. Healthcare administrators saw it as a financial cost center and a compliance challenge, focusing on meeting stringent data-protection and safety requirements imposed by law and regulation. These are not complementary perspectives that naturally synthesize; they are orthogonal worldviews requiring active translation. Our solution suggests including:

1. *Boundary spanners*: Individuals with hybrid expertise who are able to translate between domains and disciplines, a role well-theorized in the organizational and science studies literatures.^12^^,^^13^
2. *Collaborative artifacts*: A code of conduct that serves as much to align understanding as to establish rules on how to achieve the set goals. Such documents function as “boundary objects”,^12^ artifacts plastic enough to accommodate different interpretations across stakeholder groups yet robust enough to maintain a common identity and enable coordination.
3. *Iterative refinement*: Various rounds of workshops to surface and resolve conceptual mismatches and build a shared understanding.

This translation work is not preliminary to the “*real*” technical work. It is the foundation that makes the technical work possible. Federated learning projects that skip this phase are not saving time. They are heading toward failure.

## Provocations for the field

Based on our experience, we offer 4 provocations to advance federated learning from promise to practice:

1. *Stop pretending federated learning is primarily about privacy*
  The goal is to enable collaboration between entities, which requires mutual trust. Privacy preservation is only a means toward the end. This highlights the need to design for trust-building, not just data protection.
2. *Acknowledge that federated learning may increase rather than decrease inequality*
  Resource requirements for participation are substantial. Without deliberate intervention, federated networks will concentrate power among already-powerful institutions.
3. *Recognize that current evaluation metrics are misleading*
  Comparing federated versus centralized model performance ignores coordination costs, governance complexity, and implementation barriers. A holistic evaluation framework would capture total system cost and complexity while evaluating the performance through domain expertise to cover cost, complexity, performance, ethics, safety, and governance.
4. *Accept that human factors determine success*
  The limiting factor is not algorithm design or network architecture; it is the capacity of human organizations to collaborate across institutional, disciplinary, and national boundaries.

## It is all about evolution, not revolution

Federated learning represents an evolutionary step in health data use and collaboration, not the revolutionary leap its promoters claim. It solves some problems related to data use and privacy preservation while creating other problems, such as coordination complexity, governance challenges, data heterogeneity, and resource disparities. Success requires abandoning techno-utopian visions of frictionless global collaboration and embracing the messy reality of human organizations struggling to work together. It means investing more in partnership and governance than in GPUs, more in translation than in transmission, and more in trust than in technology. The *Nordic-Baltic Federated Health Data Network* was an experiment that succeeded because we overcame technical challenges as well as recognized and addressed human challenges that others would not. Our organizational achievement, getting 9 institutions across 5 countries to collaborate effectively is what matters. Importantly, identifying these challenges should not be mistaken for an endorsement of centralization. The very limitations we describe, governance gaps, coordination costs, and trust deficits are, in many cases, amplified rather than resolved by centralized alternatives. Federated learning remains the most promising architecture for privacy-respecting, multi-institutional collaboration. We argue that its success depends on investing as seriously in human infrastructure as in technical infrastructure.

The field stands at a crossroads. Continuing down the current path, focusing mainly on the technical optimization and architectural refinement in federated learning, will remain an academic exercise, impressive in papers but impractical in practice. Alternatively, acknowledging the primacy of human and organizational factors in federated learning could enable previously impossible collaborations. The choice is not technical, but philosophical. The key question is whether we continue to assume that increasingly sophisticated algorithms can resolve those issues, which are essentially human challenges, or whether we acknowledge that federated learning is ultimately shaped by people, policies and power, and technology only serves as the enabling platform. We offer a possible answer, but is the field ready to confront it?

## Data Availability

No new datasets were generated or analyzed for this work.
